# EAONO/JOS classification for acquired cholesteatoma: evaluating the impact of the number of affected sites on treatment and outcomes

**DOI:** 10.1007/s00405-023-07996-w

**Published:** 2023-05-23

**Authors:** B. Renner, A. Vasconcelos Craveiro, M. Balk, M. Allner, M. Sievert, S. K. Mueller, K. Mantsopoulos, H. Iro, R. Rupp, J. Hornung, A. O. Gostian

**Affiliations:** Department of Otolaryngology, Head and Neck Surgery, University Hospital of Erlangen, Erlangen, Germany

**Keywords:** Cholesteatoma, EAONO/JOS classification, Tympanoplasty

## Abstract

**Purpose:**

The European and Japanese system for cholesteatoma classification proposed an anatomical differentiation in five sites. In stage I disease, one site would be affected and in stage II, two to five. We tested the significance of this differentiation by analyzing the influence of the number of affected sites on residual disease, hearing ability and surgical complexity.

**Methods:**

Cases of acquired cholesteatoma treated at a single tertiary referral center between 2010-01-01 and 2019-07-31 were retrospectively analyzed. Residual disease was determined according to the system. The air–bone gap mean of 0.5, 1, 2, 3 kHz (ABG) and its change with surgery served as hearing outcome. The surgical complexity was estimated regarding the Wullstein’s tympanoplasty classification and the procedure approach (transcanal, canal up/down).

**Results:**

513 ears (431 patients) were followed-up during 21.6 ± 21.5 months. 107 (20.9%) ears had one site affected, 130 (25.3%) two, 157 (30.6%) three, 72 (14.0%) four and 47 (9.2%) five. An increasing number of affected sites resulted in higher residual rates (9.4–21.3%, p = 0.008) and surgical complexity, as well poorer ABG (preoperative 14.1 to 25.3 dB, postoperative 11.3–16.8 dB, p < 0.001). These differences existed between the means of cases of stage I and II, but also when only considering ears with stage II classification.

**Conclusion:**

The data showed statistically significant differences when comparing the averages of ears with two to five affected sites, questioning the pertinence of the differentiation between stages I and II.

**Supplementary Information:**

The online version contains supplementary material available at 10.1007/s00405-023-07996-w.

## Introduction

The Japanese Otological Society (JOS) published in 2008 a classification system for middle ear cholesteatoma [1], which was reviewed in 2010 [2] and 2015 [3]. The European Academy of Otology and Neurotology (EAONO) presented its own system in 2015, which also featured the disease recidivism [4]. Seeking an international uniformization, both parts joined efforts and released in 2016 the EAONO/JOS consensus system [5]. Five anatomical sites were categorized: supratubal recess (S1), sinus tympani (S2), tympanic cavity (T), attic (A) and mastoid (M). Four stages were defined. In stage I, the cholesteatoma affects only one site, with two possibilities: “A” referring to a pars flaccida cholesteatoma (PFC) or “T” for a pars tensa cholesteatoma (PTC). In stage II, two to five sites are affected. The presence of extracranial or intracranial complications results respectively in a stage III or IV classification. So stages I and II only refer to disease extension and to some extent localization, excluding any complications. Recidivism was the only described treatment outcome and a differentiation between residual disease (incomplete surgical removal with no contact to the tympanum) and recurrence (after complete surgical removal, reformation of the retraction pocket and new cholesteatoma) was made. An international validation study of nine centers and 1482 cases demonstrated statistically significant differences in 5 years residual and recurrence rates between stages I and II, respectively 3–13% and 4–10% [6]. Another retrospective single-center study with 34 patients with PFC found a relation between better postoperative audiological outcome and three factors: lower stage (stages I, II and III), better condition of the stapes and a better development of the mastoid cells [7]. A single-center cohort of 125 patients with retraction pocket cholesteatomas (RPC) showed no direct correlation between stage and recidivism. However, involvement of S1 led to a more frequent recidivism. Higher rates of residual cholesteatoma were associated with a defect greater than half of the posterior auditory canal and patients younger than 15 years [8]. In addition, a single-center analysis of 290 patients with a mean follow-up of 4 years showed that a higher disease stage resulted in worse hearing outcome and more frequent canal wall down procedures, while a correlation between stage and recidivism was not registered [9]. The available research considering the EAONO/JOS consensus shows conflicting findings about the relationship between stage and different pathophysiological/epidemiological characteristics, as well as treatment outcomes. Moreover, these studies analyzed several characteristics inherent to the disease that were not featured in the EAONO/JOS proposal, as hearing ability, age, ossicle state, among others. To address these questions, further developing a consensus about cholesteatoma, but also to uniformize the report of otologic data, the International Otology Outcome Group (IOOG) was established in 2017 (www.IOOG.net). Our study intended to contribute for these purposes. The primary objective was to assess the pertinence of the differentiation between stages I and II. For this purpose, the impact of the number of affected sites on residual rate, hearing ability and surgical complexity was analyzed. We hypothesized that an increasing number of affected sites would result in more frequent residual rate, poorer hearing outcomes and greater surgical complexity. We assumed that these differences would exist between cases with one affected site (stage I) and two to five affected sites (stage II), but also within stage II. As only recidivism was discussed in the EAONO/JOS system, the secondary objective was to demonstrate the relevance of the hearing outcomes and surgical procedure in the evaluation of cholesteatoma and its treatment.

## Methods

The medical records of patients that underwent a tympanoplasty (TPL) due to cholesteatoma at the Department of Otorhinolaryngology and Head and Neck Surgery, Friedrich-Alexander-University Erlangen-Nürnberg (FAU), Germany, a tertiary referral medical center, between 2010-01-01 and 2019-07-31 were retrospectively analyzed and classified according to the EAONO/JOS system (5). The present study was performed in fulfillment of the requirements for obtaining the degree “Dr. med.”, approved by the Local Ethics Committee (Nr. 371_20 Bc) and carried out according to the Declaration of Helsinki. The following inclusion criteria were applied: acquired cholesteatoma; disease stages I and II; complete medical records including surgery reports, documented pre- and post-surgery, bone and air conduction audiograms; minimum follow-up of 3 months. The considered exclusion criteria were: congenital and unclassifiable cholesteatoma; presence of complications (disease stages III and IV); prior TPL for cholesteatoma treatment in the concerning ear. In patients with bilateral cholesteatoma, each ear represented an independent case. Recidivism was differentiated in residual and recurrent disease according to the EAONO/JOS classification. Hearing ability is presented by the mean pure-tone air-bone-gap (ABG) of the frequencies 0.5, 1, 2 and 3 kHz as described by the American Academy of Otolaryngology—Head and Neck Surgery Foundation (AAO-HNS) [10]. Pre- and postoperative ABG were analyzed, as well the difference between both. The type of Wullstein’s TPL and the surgical approach were considered as indicators of the surgical complexity. The surgical approach was classified in increasing difficulty as: transcanal (TC), canal wall up (CWU) and canal wall down (CWD). There was a differentiation of the CWD procedures: without reconstruction, simply CWD, or with reconstruction of the posterior canal wall using auricular or tragal cartilage (CWR, in the same surgery). The influence of the number of affected sites (independent variable) on recidivism, hearing ability and surgical complexity was analyzed.

### Statistical analyses

Continuous variables were tested for normal distribution via the Kolmogorov Smirnov Test and by QQ-Plots and histograms. These variables are presented with the mean ± 1 standard deviation and range (min–max). Nominal variables are presented as absolute and relative frequencies (N/%). The means of the parameters recidivism, hearing ability and surgical complexity according to the number of affected sites are always the subject of the different comparisons, even when this is not explicitly written (to simplify the text). Changes of the pre- to postsurgical ABG were compared with a dependent t-test. Differences in ABG between groups were analyzed with a between-subjects ANOVA with the main effect “number of affected sides”. To test the hypothesis that the patients with more affected sides showed higher ABG, linear contrasts were performed. The partial Eta^2^ was reported as the effect size of the main effect “number of affected sides” of the ANOVA. Here, an Eta^2^_p_ of 0.01 equals a small effect, 0.06 a medium effect and 0.14 a strong effect. Bonferroni correction was performed for all explorative pairwise post-hoc comparisons. Differences between groups regarding nominal variables were tested with cross tables and the Chi^2^-test. If cross tables with more dimension than 2 × 2 were significant, effects were further investigated with 2 × 2 cross tables for pairwise comparisons. Effect sizes are reported for t-tests in terms of r and for the Chi^2^-test with phi (2 × 2 cross tables) or Cramer’s V (> 2 × 2 cross tables). An r, phi or Cramer’s V of 0.1 displays a small effect, 0.3 represents a moderate effect and 0.5 a strong effect. A p ≤ 0.05 was considered as statistically significant, but P-values ≤ 0.05 marked with an asterisk (*) should be interpreted as only a trend towards significance after correction for multiple comparisons. Data analysis was performed with IBM SPSS Statistics Version 28.0. All statistic computations are shown in the publication supplement.

## Results

Between 2010-01-01 and 2019-07-31, a total of 2005 TPLs to treat cholesteatoma were performed. Out of these, 513 TPLs (right ears 254/49.5%, female 185/35.9%) met the inclusion/exclusion criteria. 41 patients (female 10/24.4%) were treated on both sides, so that 472 subjects (female 175/37.1%) were analyzed. The mean age was 39.0 ± 20.7 years (4–82 years) and the mean follow-up time 21.6 ± 21.5 months (3–112 months). A total of 107/20.9% ears showed one affected site, 130/25.3% two sites, 157/30.6% three, 72/14.0% four and 47/9.2% five (Table 1). All surgical procedures were performed by ten experienced otologic surgeons.

**Table 1:** Demographic und clinical data of the patients

Number of cases	513
Number of patients	472
Patients treated both sides (N/%)	41/8.7
Age (years, mean ± SD, min–max)	39.0 ± 20.7, 4–82
Follow-up (months, mean ± SD, min–max)	21.6 ± 21.5, 3–112
Side (N (%))	
Right	254 (49.5)
Left	259 (50.5)
Gender (N (%))	
Male	529 (64.1)
Female	184 (35.9)
Number of affected sites (N(%))	
One	107 (20.9)
Two	130 (25.3)
Three	157 (30.6)
Four	72 (14.0)
Five	47 (9.2)

**Fig. 1:**
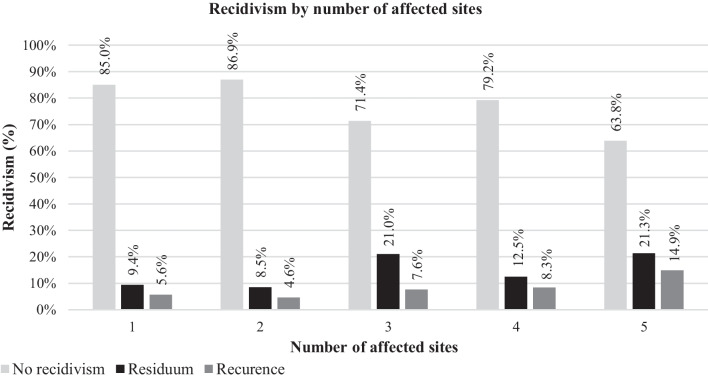
Cholesteatoma recidivism (%) according to the number of affected sites considering the EAONO/JOS classification. The impact of the number of affected sites was statistically significant on residual disease (residuum) (p = 0.008), but not on recurrence (p = 0.196). However, the results concerning the recurrence rate are conditioned by the brief follow-up (average of 21.6 months and minimum of 3)

A total of 403/78.6% ears developed no recidivism, 73/14.2% developed a residual disease and 37/7.2% a recurrence (Fig. 1). The rate of residual disease increased with the number of involved sites (p = 0.008, Cramer’s V = 0.163). Post-hoc comparisons showed that on average ears with one affected site developed less often a residual disease compared to the cases with three (9.3% vs. 21.0%, p = 0.012*, phi = 0.155) and five (21.3%, p = 0.043*, phi = 0.163). Similarly, cases with two affected sites accounted for less residual disease compared to three (8.5% vs 21.0%) (p = 0.003, phi = 0.065) and five (21.3%, p = 0.020*, phi = 0.175). During the short follow-up time, the number of affected sites did not significantly influence the recurrence rates (p = 0.196, Cramer’s V = 0.109).

**Fig. 2:**
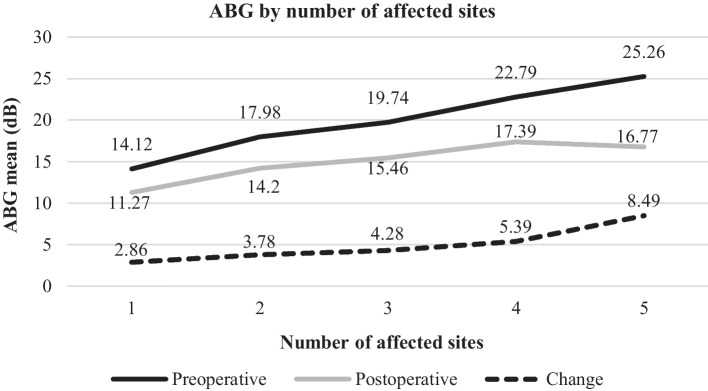
Means of the preoperative, postoperative and change of the ABG due to surgery according to the number of affected sites by cholesteatoma considering the EAONO/JOS classification. The preoperative and postoperative differences were statistically significant, but not the change. ABG—mean pure-tone air-bone-gap of the frequencies 0.5, 1, 2 and 3 kHz

The overall preoperative ABG was 19.1 ± 11.7 (0.3–55.0) dB, which improved postoperatively to 14.6 ± 9.5 (0.6–101.0) dB by 4.4 ± 11.29 (-46.0–45.3) dB (p < 0.001, r = 0.363, Fig. 2). The main effect “number of affected sites” led to statistically significant increase of the preoperative ABG (p < 0.001, Eta^2^_p_ = 0.080; linear contrast: p < 0.001) and postoperative ABG (p < 0.001, Eta^2^_p_ = 0.045; linear contrast: p < 0.001). The change of the ABG was not statistically significant (p = 0.057, Eta^2^_p_ = 0.018; linear contrast: p = 0.003). Regarding the preoperative ABG, a significant difference was found between the means of cases with one affected site and three/four/five (p < 0.001), as well when comparing ears with two affected sites to four (p = 0.004) or five (p < 0.001) and between cases with three affected sites and five (p = 0.004). A trend to significance was present between ears with one and two affected sites (p = 0.007*). Regarding the postoperative ABG, there was a significant difference between cases with one and three/four (p < 0.001) or five (p = 0.003) affected sites. A trend to significance was registered between ears with one involved site and to two (p = 0.024*), as well when comparing the means of two and four affected sites (p = 0.019*).

**Fig. 3:**
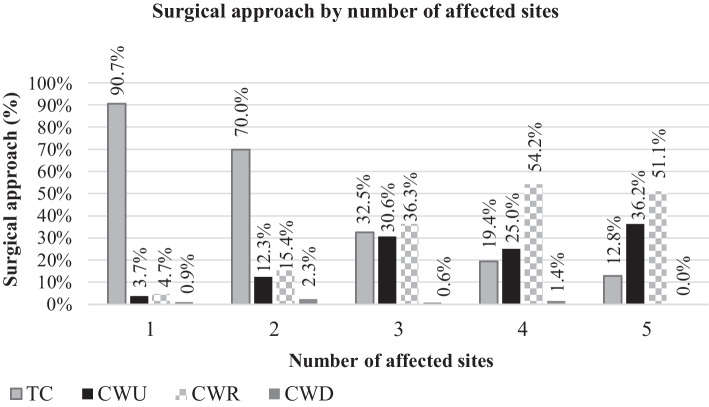
Performed surgical approach considering the number of affected sites with cholesteatoma according to the EAONO/JOS classification. *TC* transcanal, *CWU* canal wall up, *CWR* canal wall down with reconstruction, *CWD* canal wall down without reconstruction

Looking at the surgical approach, TC was performed in 259/50.5% of the cases, CWU in 103/20.1%, CWR in 145/28.3% and CWD in 6/1.2% (Fig. 3). In general, a higher number of involved sites required a more complex surgical approach (p < 0.001, Cramer’s V 0.337). An increasing number of affected sites resulted in less frequent TC (p < 0.001, Cramer’s V = 0.565). Patients with one and two affected sides received TC respectively in 90.7% and 70.0% of the cases, while TC was less often performed in patients with three (32.5%), four (19.4%) and five sites (12.8%) involved. A significant difference was computed between ears with one affected site and ears with more than one affected site (p < 0.001, phi = 0.254), as well between cases with two and three to five sites (p < 0.001, phi = 0.254). Between ears with three sites and four (p ≤ 0.042*, phi = 0.134) or five (0.008*, phi = 0.185) only a trend was shown. The opposite was observed for CWU (p < 0.001, Cramer’s V = 0.287) and CWR (p < 0.001, Cramer’s V = 0.397), i.e. these approaches were more frequently performed the more sites were involved. Ears with one and two affected sites required a CWU in 3.7% and 12.3% of the cases (CRW 4.7% and 15.4%). On the other hand, when three, four or five sites were affected, CWU was performed in 30.6%, 25.0%, 35.2% and CWR in 36.3%, 54.2% and 51.1% of the cases, respectively. Considering CWU, a trend to significance was shown when comparing ears with one affected site and two (p = 0.018*, phi ≥ 0.153) and two affected sites and four (p = 0.021*, phi = 0.162). A statistically significant difference was present when comparing cases with two affected sites and three (p < 0.001, phi = 0.331), four (p < 0.001, phi = 0.318) or five (p < 0.001, phi = 0.435). For CWR, a trend to significance was found between cases with one affected site and two (p = 0.008*, phi = 0.174), as well between three and four (p = 0.011*, phi = 0.168). The data showed a significant difference between ears with one affected site and three (p < 0.001, phi = 0.366), four (p < 0.001, phi = 0.564) and five (p < 0.001, phi = 0.546), as well when comparing cases with two involved sites and three (p < 0.001, phi = 0.235), four (p < 0.001, phi = 0.408) and five (p < 0.001, phi = 0.365). No trend or significance was found considering only CWD (without reconstruction), probably because of the low number of such cases.

**Fig. 4:**
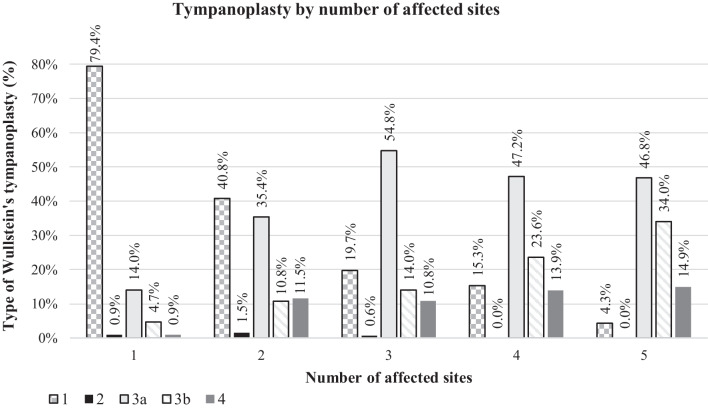
Type of performed Wullstein’s tympanoplasty considering the number of affected sites with cholesteatoma according to the EAONO/JOS classification

TPL type I according to Wullstein was performed in 182/35.5% of all cases, type II in 4/0.8%, type IIIa in 203/39.6%, type IIIb in 74/14.4% and type IV in 50/9.7%. No type V surgery was conducted. (Fig. 4) An increasing number of affected sites demanded a more complex TPL (p < 0.001, Cramer’s V = 0.557). TPL I was more common when one site was affected (79.4%) and decreased to 4.3% in ears with five affected sites (p < 0.001, Cramer’s V = 0.526). A significant difference was identified between ears with one affected site and two (p < 0.001, phi = 0.390), three (p < 0.001, phi = 0.590), four (p < 0.001, phi = 0.631) and five (p < 0.001, phi = 0.698), as well between cases with two affected sites and three (p < 0.001, phi = 0.230), four (p < 0.001, phi = 0.262) and five (p < 0.001, phi = 0.348). Comparing three sites to five, a trend towards significance was shown (p = 0.011*, phi = 0.177). TPL IIIa also differed significantly according to the number of affected sites (p < 0.001, Cramer’s V = 0.306). It was less frequently performed in patients with one affected site (14.0%), rising to 35.4% by two affected sites and further increasing when three to five sites were involved (46.8–54.8%). A significant difference was present when comparing cases with one affected site to two (p < 0.001, phi = 0.243), three (p < 0.001, phi = 0.412), four (p < 0.001, phi = 0.365) and five (p < 0.001, phi = 0.353). Additionally, the difference between ears with two a three affected sites was statistically significant (p = 0.001, phi = 0.194). The frequency of TPL IIIb (34.0%) was higher in cases with five affected sites and decreased with less affected sites (4.7–23.6%). Overall, these differences were statistically significant (p < 0.001, V = 0.239). Concretely, significant differences were found between cases with one and four (p < 001, phi = 0.283) or five (p < 001, phi = 0.394) affected sites, as well between ears with two and five affected sites (p < 0.001, phi = 0.274) and when comparing cases with three and five involved sites (p = 0.002, phi = 0.217). A trend to significance was found between cases with one and three affected sites (p = 0.014*, phi = 0.151), as well between two and four (p = 0.015*, phi = 0.171). The number of affected sites also influenced the frequency how TPL IV was performed (p = 0.012, Cramer’s V = 0.159). Here, cases with one affected site received on average the procedure systematically less often (0.9%) than others (11.5–14.9%). The analysis showed the following significances: comparing cases with one affected site to two (p = 0.001, phi = 0.210), one to three (p = 0.002, phi = 0.193), one to four (p < 0.001, phi = 0.264) and one to five (p < 0.001, phi = 0.290).

## Discussion

513 cases of acquired cholesteatoma, treated between 2010-01-01 and 2019-07-31, in a single tertiary center, were classified according to the EAONO/JOS consensus and retrospectively analyzed. On average an increasing number of affected sites led to higher residual rates, poorer preoperative and postoperative ABG and higher complexity regarding the surgical approach and TPL according to the Wullstein’s classification. The effect of surgery on the ABG was independent of the number of affected sites, encouraging outcome that reflects the excellency of the modern treatment, no matter how extensive the disease might be. In fact, even if not statistically significant, a worse starting ABG resulted on average in a greater improvement, as the change of the ABG increased with the number of affected sites.

This is the first study to focus on the number of affected sites defined in the EAONO/JOS system (stages I and II) as an independent variable, excluding any complications (stages III and IV). Statistically significant differences were demonstrated between the means of cases with one and two to five affected sites, reflecting a difference between stages I and II. However, these differences were also present between ears both in stage II, with greater relevance when comparing cases with two involved sites and cases with three to five, questioning the significance of the differentiation between stages I and II, the main objective of our study. The confirmation of the influence of the number of affected sites on the hearing ability and surgical complexity met our secondary objective.

Ears with one affected site and two presented a residual rate under 10%, while the rest of the cases had a superior one. Regarding the preoperative ABG, statistically significant differences were seen between cases with one affected site and three to five, but also between ears with two affected sites and four or five and even between three and five sites. Looking at the postoperative ABG, statistically significant differences were found between cases with one affected site and cases with three to five. Similarly, the differences regarding the surgical approach were also evident between cases with one affected site and all others, but also between two affected sites and all others. Furthermore, the distribution of the tympanoplasties according to the Wullstein’s classification also did not support the distinction into stages I and II. For type I, IIIa and IV there were statistically significant differences between cases with one affected site and all others, but for instance in type I these differences were also present between ears with two affected sites and all others.

As our independent variable was the number of affected sites, the comparison of our results with the ones of other publications is challenging, but possible to some extent. James et al. demonstrated a higher residual rate between stages I and II [6], contrary to Angeli et al. [8] and Ardiç et al. [9]. This last publication and the one from Fukuda et al. [7] correlated a higher disease stage with poorer audiological results. However, none of the two studies specified a difference between stage I and II. Moreover, both publications considered an ABG of ≤ 10 or ≤ 20 dB as successful outcomes, therefore not allowing a meaningful comparison with our results.

The EAONO/JOS classification discusses recidivism as a treatment outcome, which we also acknowledge, as extremely important. Nevertheless, the hearing ability can also be considered as equally relevant. A future revision of the EAONO/JOS system could also feature the hearing ability and set indications how to evaluate it, for instance following the AAO-HNS recommendations [10].

Focusing on our data, grouping cases with one affected site and two in stage I and cases with three to five in stage II would be an option to consider. A group of international surgeons responded to the EAONO/JOS classification with an alternative system called ChOLE [11], which could eventually better correlate with our findings. In the ChOLE, four disease stages based only on the cholesteatoma extension were described. In the first two, only the middle ear is affected, in this aspect similar to the EAONO/JOS classification. In stage 1a, the epitympanic space is affected and in 1b, the epitympanic space and the sinus tympani. In stage 2a, the cholesteatoma would extend further to the attic and antrum and in stage 2b obligatory to the supratubal recess, optionally to the protympanum and/or sinus tympani. However, returning to our results, there were also significant differences between cases with one and two affected sites and between ears with three, four or five, raising the question if the classification should not always specify which sites are affected. This was suggested in the STAMCO system from a multicenter nationwide Dutch study group, which intended to further develop the EAONO/JOS classification [12]. The EAONO/JOS group responded by welcoming this Dutch study, as the progressive evolution of the classification based in international consensus is itself the main goal [12].

As expected, and considering our data, the number of affected sites also correlated with the surgical approach. Another publication showed that a CWD was performed more often with increasing stage (p < 0.001) [9]. In our population, a statistically significant influence of the number of affected sites on the performed TPL according to the Wullstein’s classification was registered, which reflects indirectly the state of the ossicles. Fukuda et al. [7] reported a better state of the stapes for lower disease stages, without directly comparing the different stages. The former JOS classification of 2015 [3], the already mentioned ChOLE [11] and STAMCO [12] also featured the ossicles state. The IOOG proposed a classification of tympanomastoid surgery, featuring for instance the type of intervention and state of different anatomical structures, although not the hearing outcomes [13]. So, an international consensus statement could consider all factors that can apply to tympanomastoid surgery in general, like the type of procedure, state and reconstruction of the ossicles, hearing outcomes, among others. The EAONO/JOS system would continue to approach aspects exclusively related to cholesteatoma, namely its definition, classification and recidivism.

The presented study is limited due to the inevitable bias inherent to a retrospective study design of one single-center. However, when interpreting the results, it should be taken into consideration that only ten experienced surgeons were involved, as well as the considerably high number of included patients and the homogeneity of the performed procedures, according to the institution surgical guidelines. Another critical point is the follow-up time, with an average of 21.6 months and a minimum of 3. In such a brief period, conclusions regarding recurrence rates, if any, should be drawn with caution. The fact that the patients ages ranged from 4 to 82 years should also be pointed out, as cholesteatoma differs between children and adults. In fact, a publication regarding the EAONO/JOS consensus showed that cholesteatoma is more aggressive and relapses more frequently in pediatric age (defined as < 16 years old) [14].

Our results indicate that the existing classification is certainly useful but may be improved in clinical application and in reflecting the cases to be treated. This is especially true, when considering the study objectives chosen here and that the classification of acquired cholesteatoma should be widely used for preoperative preparation, intraoperative approach and postoperative care. For this, further multi-center prospective trials are desirable to obtain reliable results of higher evidence.

## Conclusion

Considering the EAONO/JOS system for cholesteatoma, the data showed that the number of affected sites with disease influences the residual rate, hearing outcomes, surgical approach and type of Wullstein’s TPL. The results did not support the purposed differentiation in stages I and II. We advocate for the further development of the definition and classification of the pathology, as well of the uniformization of the description of the treatment and its outcomes. This should be based on prospective randomized trials, always seeking international consensus.

### Supplementary Information

Below is the link to the electronic supplementary material.

Supplementary file1 (DOCX 26 KB)

## Data Availability

The data supporting the study are available within the article and supplement.
