# Supplementary material for: EAONO/JOS classification for acquired cholesteatoma: evaluating the impact of the number of affected sites on treatment and outcomes
Source: Eur Arch Otorhinolaryngol. 2023 May 23;280(11):4827–34. doi: 10.1007/s00405-023-07996-w (PMC10562301; doi:10.1007/s00405-023-07996-w)
Supplement: Supplementary file 1 — Supplementary file1 (DOCX 26 KB) [file 405_2023_7996_MOESM1_ESM.docx]

|  | Number of affected sides | | | | |  |
| --- | --- | --- | --- | --- | --- | --- |
|  | 1 | 2 | 3 | 4 | 5 |  |
| Recurrence / Residual disease / none |  |  |  |  |  | **p = 0.006**,  V = 0.144 |
| Recurrence |  |  |  |  |  | p = 0.196,  V = 0.109 |
| Residual disease | vs. | p = 0.812,  phi = 0.015 | **p = 0.012***,  phi = 0.155 | p = 0.502,  phi = 0.050 | **p = 0.043***,  phi = 0.163 | **p = 0.008**,  V = 0.163 |
|  |  | vs. | **p = 0.003,** phi = 0.065 | p = 0.357,  phi = 0.065 | **p = 0.020***,  phi = 0.175 |  |
|  |  |  | vs. | p = 0.122,  phi = 0.102 | p = 0.970  phi = 0.003 |  |
|  |  |  |  | vs. | p = 0.201,  phi = 0.117 |  |

Supplement

Table 1: Statistical comparison of no recidivism, recurrent and residual disease regarding the number of affected sides with cholesteatoma. P-values ≤ 0.05 are highlighted in bold. Results not significant after correction for multiple comparisons are marked with *.

|  | Number of affected sides | | | | |  |
| --- | --- | --- | --- | --- | --- | --- |
|  | 1 | 2 | 3 | 4 | 5 | ANOVA |
| ABG pre. | vs. | **p = 0.007***,  r = 0.174 | **p < 0.001**,  r = 0.237 | **p < 0.001**,  r = 0.355 | **p < 0.001**,  r = 0.426 | **p < 0.001**,  Eta_p_^2^ = 0.080 |
|  |  | vs. | p = 0.191,  r = 0.077 | **p = 0.004,**  r = 0.203 | **p < 0.001**,  r = 0.284 |  |
|  |  |  | vs. | p = 0.067,  r = 0.121 | **p = 0.004**,  r = 0.199 |  |
|  |  |  |  | vs. | p = 0.251,  r = 0.106 |  |
| ABG post. | vs. | **p = 0.024***,  r = 0.146 | **p < 0.001**,  r = 0.208 | **p < 0.001**,  r = 0.269 | **p = 0.003**,  r = 0.238 | **p < 0.001**,  Eta_p_^2^ = 0.045 |
|  |  | vs. | p = 0.215,  r = 0.073 | **p = 0.019***,  r = 0.165 | p = 0.078,  r = 0.133 |  |
|  |  |  | vs. | p = 0.132,  r = 0.100 | p = 0.346,  r = 0.066 |  |
|  |  |  |  | vs. | p = 0.722,  r = 0.033 |  |
| ABG change |  |  |  |  |  | p = 0.057,  Eta_p_^2^ = 0.018ᵀ |

Table 2: Statistical comparison of air-bone gap mean of 0.5, 1, 2, 3 kHz (ABG) in pre-, postoperative and their change with surgery regarding the number of affected sides with cholesteatoma. P-values ≤ 0.05 are highlighted in bold. Results not significant after correction for multiple comparisons are marked with *.

|  | Number of affected sides | | | | |  |
| --- | --- | --- | --- | --- | --- | --- |
|  | 1 | 2 | 3 | 4 | 5 |  |
| Surgical Approach |  |  |  |  |  | **p < 0.001**,  V = 0.337 |
| Transcanal | vs. | **p < 0.001**,  phi = 0.254 | **p < 0.001**,  phi = 0.575 | **p < 0.001**,  phi = 0.719 | **p < 0.001**,  phi = 0.762 | **p < 0.001**,  V = 0.565 |
|  |  | vs. | **p < 0.001**, phi = 0.374 | **p < 0.001**,  phi = 0.485 | **p < 0.001**,  phi = 0.508 |  |
|  |  |  | vs. | **p = 0.042***, phi = 0.134 | **p = 0.008***,  phi = 0.185 |  |
|  |  |  |  | vs. | p = 0.341,  phi = 0.087 |  |
| Canal wall up | vs. |  |  |  |  | **p < 0.001**,  V = 0.287 |
|  |  | **p = 0.018*,**  phi = 0.153 | **p < 0.001**,  phi = 0.331 | **p < 0.001**,  phi = 0.318 | **p < 0.001**,  phi = 0.435 |  |
|  |  | vs. | **p < 0.001**, phi = 0.218 | **p = 0.021***,  phi = 0.162 | **p < 0.001**,  phi = 0.271 |  |
|  |  |  | vs. | p = 0.387,  phi = 0.057 | p = 0.470,  phi = 0.051 |  |
|  |  |  |  | vs. | p = 0.191,  phi = 0.120 |  |
| Canal wall reconstruction | vs. | **p = 0.008*,**  phi = 0.174 | **p < 0.001**,  phi = 0.366 | **p < 0.001**,  phi = 0.564 | **p < 0.001**,  phi = 0.546 | **p < 0.001**,  V = 0.397 |
|  |  | vs. | **p < 0.001**, phi = 0.235 | **p < 0.001**,  phi = 0.408 | **p < 0.001**,  phi = 0.365 |  |
|  |  |  | vs. | **p = 0.011***, phi = 0.168 | p = 0.070,  phi = 0.127 |  |
|  |  |  |  | vs. | p = 0.740,  phi = 0.030 |  |
| Canal wall down |  |  |  |  |  | p = 0.648,  V = 0.070 |

Table 3: Statistical comparison of surgical approach regarding the number of affected sides with cholesteatoma. P-values ≤ 0.05 are highlighted in bold. Results not significant after correction for multiple comparisons are marked with *.

|  | Number of affected sides | | | | |  |
| --- | --- | --- | --- | --- | --- | --- |
|  | 1 | 2 | 3 | 4 | 5 |  |
| Wullstein‘s classification |  |  |  |  |  | **p < 0.001**,  V = 0.557 |
| I | vs. | **p < 0.001**,  phi = 0.390 | **p < 0.001**,  phi = 0.590 | **p < 0.001**,  phi = 0.631 | **p < 0.001**,  phi = 0.698 | **p < 0.001**,  V = 0.526 |
|  |  | vs. | **p < 0.001**, phi = 0.230 | **p < 0.001**,  phi = 0.262 | **p < 0.001**,  phi = 0.348 |  |
|  |  |  | vs. | p = 0.417,  phi = 0.054 | **p = 0.011***,  phi = 0.177 |  |
|  |  |  |  | vs. | p = 0.060,  phi = 0.173 |  |
| IIIa | vs. |  |  |  |  | **p < 0.001**,  V = 0.306 |
|  |  | **p < 0.001**,  phi = 0.243 | **p < 0.001**,  phi = 0.412 | **p < 0.001**,  phi = 0.365 | **p < 0.001**,  phi = 0.353 |  |
|  |  | vs. | **p = 0.001**, phi = 0.194 | p = 0.099,  phi = 0.116 | p = 0.168,  phi = 0.104 |  |
|  |  |  | vs. | p = 0.288,  phi = 0.070 | p = 0.337,  phi = 0.067 |  |
|  |  |  |  | vs. | p = 0.965,  phi = 0.004 |  |
| IIIb |  |  |  |  |  | **p < 0.001**,  V = 0.239 |
|  | vs. | p = 0.085,  phi = 0.112 | **p = 0.014***,  phi = 0.151 | **p < 0.001**,  phi = 0.283 | **p < 0.001**,  phi = 0.394 |  |
|  |  | vs. | p = 0.409, phi = 0.049 | **p = 0.015*,**  phi = 0.171 | **p < 0.001**,  phi = 0.274 |  |
|  |  |  | vs. | p = 0.073,  phi = 0.119 | **p = 0.002**,  phi = 0.217 |  |
|  |  |  |  | vs. | p = 0.214,  phi = 0.114 |  |
| IV |  |  |  |  |  | **p = 0.012**,  V = 0.159 |
|  | vs. | **p = 0.001,**  phi = 0.210 | **p = 0.002**,  phi = 0.193 | **p < 0.001**,  phi = 0.264 | **p < 0.001**,  phi = 0.290 |  |
|  |  | vs. | p = 0.849, phi = 0.011 | p = 0.627,  phi = 0.034 | p = 0.550,  phi = 0.045 |  |
|  |  |  | vs. | p = 0.505,  phi = 0.044 | p = 0.448,  phi = 0.053 |  |
|  |  |  |  | vs. | p = 0.878,  phi = 0.014 |  |

Table 4: Statistical comparison of Wullstein’s classification regarding the number of affected sides with cholesteatoma. P-values ≤ 0.05 are highlighted in bold. Results not significant after correction for multiple comparisons are marked with *.
